# Epigallocatechin Gallate-Gold Nanoparticles Exhibit Superior Antitumor Activity Compared to Conventional Gold Nanoparticles: Potential Synergistic Interactions

**DOI:** 10.3390/nano9030396

**Published:** 2019-03-08

**Authors:** Suhash Reddy Chavva, Sachin Kumar Deshmukh, Rajashekhar Kanchanapally, Nikhil Tyagi, Jason William Coym, Ajay Pratap Singh, Seema Singh

**Affiliations:** 1 Department of Oncologic Sciences, Mitchell Cancer Institute, University of South Alabama, Mobile, AL 36604, USA; srchavva@health.southalabama.edu (S.R.C.); skdeshmukh@health.southalabama.edu (S.K.D.); rkanchanapally@health.southalabama.edu (R.K.); nikhiltyagi22@gmail.com (N.T.); 2 Department of Chemistry, University of South Alabama, Mobile, AL 36688, USA; jwcoym@southalabama.edu; 3 Department of Biochemistry and Molecular Biology, College of Medicine, University of South Alabama, Mobile, AL 36688, USA

**Keywords:** EGCG, gold nanoparticle, synergism, cancer therapy, apoptosis, NF-κB

## Abstract

Epigallocatechin gallate (EGCG) possesses significant antitumor activity and binds to laminin receptors, overexpressed on cancer cells, with high affinity. Gold nanoparticles (GNPs) serve as excellent drug carriers and protect the conjugated drug from enzymatic metabolization. Citrate-gold nanoparticles (C-GNPs) and EGCG-gold nanoparticles (E-GNPs) were synthesized by reduction methods and characterized with UV-visible spectroscopy, transmission electron microscopy (TEM), and dynamic light scattering (DLS). Cytotoxicity of citrate, EGCG, C-GNPs, and E-GNPs was evaluated by the water-soluble tetrazolium salt (WST-1) assay. Nanoparticle cellular uptake studies were performed by TEM and atomic absorption spectroscopy (AAS). Dialysis method was employed to assess drug release. Cell viability studies showed greater growth inhibition by E-GNPs compared to EGCG or C-GNPs. Cellular uptake studies revealed that, unlike C-GNPs, E-GNPs were taken up more efficiently by cancerous cells than noncancerous cells. We found that E-GNP nanoformulation releases EGCG in a sustained fashion. Furthermore, data showed that E-GNPs induced more apoptosis in cancer cells compared to EGCG and C-GNPs. From the mechanistic standpoint, we observed that E-GNPs inhibited the nuclear translocation and transcriptional activity of nuclear factor-kappaB (NF-κB) with greater potency than EGCG, whereas C-GNPs were only minimally effective. Altogether, our data suggest that E-GNPs can serve as potent tumor-selective chemotoxic agents.

## 1. Introduction

Cancer is a major public health problem worldwide [1]. It is projected that in the United States nearly 1,735,350 new cancer cases will be diagnosed and 609,640 people will die from this disease in 2018 [1]. Clearly, there is a need to tackle cancer on multiple fronts, including the development of effective prevention and therapeutic strategies. One major issue with existing cancer chemotherapeutics is their high toxicity to healthy tissues at optimally effective doses, which limits their therapeutic potential [2,3]. As an alternative, several dietary phytochemicals have emerged as molecules of choice due to their high antitumor properties and negligible toxicities [4]. However, such agents suffer from poor solubility, stability, and cellular uptake; development of approaches is required to overcome these critical barriers [5].

Epigallocatechin gallate (EGCG) is one of the most abundant and active polyphenols found in green tea. It has a wide spectrum of therapeutic properties, including anti-inflammatory, anti-obesity, antidiabetic, and antitumor effects [6,7,8]. It has been documented that EGCG has anticancer activity in many cancers, including melanoma, prostate cancer, breast cancer, and pancreatic cancer [8,9,10,11]. EGCG has also been shown to bind to laminin receptors, which are overexpressed in many types of cancer, with high affinity and specificity [12,13,14,15]. Despite its ability to act as an excellent chemopreventive, therapeutic, and targeting agent, EGCG has limitations related to stability, bioavailability, and metabolic transformations under physiological conditions [16,17,18]. Nanoparticles (structures less than 100 nm in size, in at least one dimension) serve as excellent carriers of drugs due to their small size, which provides a large functional adsorbent surface per unit mass. Specially, gold nanoparticles (GNPs) are considered a better option than other nanoparticle formulations because of their biocompatibility, ease of synthesis, chemical stability, unique optical properties, and ease of surface modification as they can bind to molecules containing –SH and –NH_2_ [19]. Their biomedical applications in chemical sensing, biological imaging, and drug delivery have been demonstrated [20,21,22]. Importantly, GNPs are also shown to confer anticancer activity by directly acting on the tumor cells and/or by impacting the tumor microenvironment [23,24,25,26,27]. Furthermore, it has been demonstrated that conjugation of ligands to GNPs by nonspecific interactions increases their stability, by protecting them from enzymatic degradation [28].

In the present study, we synthesized GNPs using conventional method (citrate-GNPs/C-GNPs) and by using EGCG as a reducing agent (E-GNPs) and compared their antitumor efficacies in a variety of established cancer cell lines. Our data demonstrate that E-GNPs have greater cancer growth inhibitory potential, compared to free EGCG or C-GNPs. This appears to be associated with their greater uptake by the cancer cells and their increased potency for inhibition of the nuclear translocation of nuclear factor-kappaB (NF-κB). Together, these findings are indicative that E-GNPs have superior anticancer activity over C-GNPs, due to synergistic interactions between EGCG and GNPs.

## 2. Materials and Methods

*Reagents:* The following reagents were obtained from different vendors: Dulbecco’s modified Eagle’s medium (DMEM) and fetal bovine serum (FBS) from Atlanta Biologicals (Lawrenceville, GA, USA); penicillin (100 units/mL), streptomycin (100 μg/mL), and trypsin-EDTA from Invitrogen, (Carlsbad, CA, USA); cell proliferation reagent, water-soluble tetrazolium salt (WST-1) and TRIzol from Roche Diagnostics (Mannheim, Germany) and Life Technologies (Carlsbad, CA, USA), respectively. PE Annexin V apoptosis detection kit was from BD Bioscience (San Diego, CA, USA); gold (III) chloride trihydrate (HAuCl_4_·3H_2_O), trisodium citrate (TSC), and citrate assay kit were from Sigma-Aldrich (St. Louis, MO, USA); nuclear extract kit was from Active Motif (Carlsbad, CA, USA); epigallocatechin gallate (EGCG) was from Medchemexpress LLC (Monmouth Junction, NJ, USA); anti-cleaved caspase 7, -cleaved caspase 3 (rabbit polyclonal), anti-BCL2, -BCL-xL, -Bax, -NF-κB/p65 (rabbit monoclonal) were from Cell Signaling Technology (Danvers, MA, USA); anti-laminin receptor antibody (MLuC5, mouse monoclonal) was from Invitrogen (Carlsbad, CA, USA); anti-rabbit and anti-mouse horseradish peroxidase (HRP)-conjugated secondary antibodies were from Santa Cruz Biotechnology (Santa Cruz, CA, USA); β-actin (mouse monoclonal) antibody was from Sigma–Aldrich; ECL plus detection kit was from Thermo Scientific (Logan, UT, USA); pGL4.32 [*luc*2P/NF-B-RE/Hygro] and pRL-TK plasmids were from Promega (Madison, WI, USA); TRIzol reagent and reverse-transcribed with high capacity cDNA reverse transcription kit were from Invitrogen and Applied Biosystems (Carlsbad, CA, USA); SYBR green master mix was from Applied Biosystems (Carlsbad, CA, USA); tris-buffered saline (TBS) and Tween 20 were from Boston Bioproducts (Ashland, MA, USA).

*Cell culture:* The human normal and cancer cell lines (MCF-10A, hTERT-HPNE [HPNE], RWPE1, MDA-MB-231, MIA PaCa, and PC3) were purchased from ATCC (Manassas, VA, USA). The normal immortalized keratinocytes (HaCaT) were obtained from the German Cancer Research Center (Heidelberg, Germany). A375SM cell line was obtained from Dr Isaiah J Fidler (University of Texas MD Anderson Cancer Center, Houston, TX, USA). All the cell lines were maintained in DMEM (GE Healthcare Life Sciences) supplemented with 10% fetal bovine serum (FBS, Atlanta Biologicals), penicillin (100 units/mL), and streptomycin (100 μg/mL) (Invitrogen) in a humidified atmosphere of 5% CO_2_ at 37 °C. Cells were intermittently tested for mycoplasma contamination at our institutional flow cytometry core facility and determined to be free from infection.

*Citrate-gold nanoparticle synthesis and characterization:* Citrate-gold nanoparticles (C-GNPs) were synthesized by reducing HAuCl_4_·3H_2_O with trisodium citrate (TSC) as reported earlier, with some modifications [21]. In brief, 10 mM solution of HAuCl_4_·3H_2_O and 1.0 wt % of TSC were prepared using deionized (DI) water. An initial volume of 1.25 mL of 10 mM HAuCl_4_·3H_2_O solution was diluted to 100 mL with DI water, and the obtained solution was brought to boiling temperature in a two-neck round-bottom flask fitted with a reflux condenser while being stirred. TSC solution (0.85 mL, 1.0 wt %) was added to the boiling solution. Boiling and stirring were continued for another 30 min after the final color change to wine red, indicating the formation of C-GNPs. After cooling to room temperature, the gold nanoparticle solution was centrifuged at 5000 rpm for 2 h. After centrifugation, the supernatant was discarded, and the centrifugate was resuspended in deionized water. The gold nanoparticles were characterized by UV-visible absorption spectroscopy (Ocean Optics FLAME-CHEM-UV-VIS, Largo, FL, USA), transmission electron microscopy (TEM, JEOL-1011, Tokyo, Japan), and dynamic light scattering (DLS) (DelsaMax Pro light scattering analyzer, Backman Coulter Inc., Atlanta, GA, USA).

*EGCG-gold nanoparticle synthesis and characterization:* EGCG-gold nanoparticles (E-GNPs) were prepared by the reduction of HAuCl_4_·3H_2_O using EGCG as the reducing agent. For this, 1.5 mM EGCG solution was prepared by dissolving a sufficient amount of EGCG in 25 mL deionized water under magnetic stirring. To this solution, 25 mL of 2 mM HAuCl_4_·3H_2_O was added dropwise at room temperature under continuous magnetic stirring. The reaction mixture immediately turned the color of red wine, indicating the formation of E-GNPs. Stirring was continued for another five minutes, and particles were washed twice with DI water by centrifugation at 5000 rpm for 2 h. Thus obtained particles were resuspended in DI water to the desired concentration and stored at 4 °C for future use. Nanoparticles were characterized by UV-visible absorption spectroscopy (Ocean Optics FLAME-CHEM-UV-VIS), TEM (JEOL-1011), and DLS (Backman Coulter Inc.).

*Estimation of citrate loading:* After synthesis of C-GNPs by citrate reduction method, the nanoparticles were centrifuged at 10,000 rpm for 2 h. After centrifugation, the supernatant was collected and the excess citrate which was not loaded in C-GNPs was estimated using a citrate assay kit (Sigma-Aldrich) according to the manufacturer’s protocol. Amount of citrate loaded in C-GNPs was calculated by subtracting the amount of unloaded citrate from the total amount of citrate used for the synthesis of C-GNPs.

*Estimation of EGCG loading:* Loading of EGCG in E-GNPs was quantified by UV-visible spectroscopy. After synthesis, E-GNPs were centrifuged at 10,000 rpm for 2 h and the supernatant was collected. The concentration of EGCG in the supernatant was quantified using UV-visible spectrophotometry, at 272 nm (λ_max_). Amount of EGCG loaded in E-GNPs was calculated by subtracting the amount of EGCG in supernatant from the total amount of EGCG used for the reaction.

*Cell viability and synergy studies:* The viability of human normal and cancerous cells after treatment with E-GNP, EGCG, C-GNP, or citrate was determined by performing WST-1 assay. In brief, 5 × 10^3^ cells were seeded in 96-well plates, cultured overnight, and treated with various concentrations of C-GNP (0–200 µg/mL), E-GNP (0–200 µg/mL), citrate (0–0.82 mM), or EGCG (0–31.8 µM). Following 96 h of treatment, cells were subjected to WST-1 assay as described earlier [29]. Since we did not observe dramatic growth inhibition of cancer cells by EGCG and GNPs, we performed synergy calculations at 20% cell growth inhibition. The combination index (CI) was calculated using Equation (1) [30]:

(1) $$\mathrm{CI}=\frac{{\left(D\right)}_{{}^{1}}}{{\left(D_{20\%}\right)}_{{}^{1}}}+\frac{{\left(D\right)}_{{}^{2}}}{{\left(D_{20\%}\right)}_{{}^{2}}}+\frac{{\left(D\right)}_{{}^{1}}\times {\left(D\right)}_{{}^{2}}}{{\left(D_{20\%}\right)}_{{}^{1}}\times {\left(D_{20\%}\right)}_{{}^{2}}},$$

where (D_20%_)_1_ and (D_20%_)_2_ are the doses of free EGCG and C-GNP, respectively, inhibiting 20% cell growth, while (D)_1_ is the dose of EGCG and (D)_2_ is the dose of GNP in E-GNP nanoformulation that gives 20% inhibition of cell growth.

*Cellular uptake studies:* RWPE1, MCF-10A, PC3, and MDA-MB-231 cells (2.5 × 10^5^ cells/well) treated with E-GNPs (50 µg/mL) or C-GNPs (50 µg/mL) were collected at various time intervals (1, 3, 6, 12, and 24 h), after washing two times with PBS. For atomic absorption spectroscopy (AAS) analysis, cells were lysed by sonication and the released nanoparticles were centrifuged, washed, diluted appropriately in 1% HNO_3_, and subjected to AAS analysis with a graphite furnace atomic absorption spectrometer (Perkin-Elmer AAnalyst 800, Waltham, MA, USA) equipped with AS 800 autosampler. A linear calibration curve between 20 and 160 ppb was made by analyzing the dilutions made from a 1000 ppm stock Au standard (Inorganic Ventures, Christiansburg, VA, USA) with 1% trace metal grade HNO_3_ in DI water. All analytical samples were in the range of the calibration curve. A 6 µL aliquot of a 0.075% Pd and 0.05% Mg(NO_3_)_2_ matrix modifier (Alfa Aesar, Ward Hill, MA, USA) was added to all the samples and standards. Sample injection volume was 20 µL. The 242.8 nm line from an Au hollow-cathode lamp was used for excitation, with a lamp current of 10 mA. The furnace temperature program was as follows: Drying at 110 °C for 30 s, followed by drying at 130 °C for 30 s, followed by ashing at 800 °C for 20 s, followed by atomization at 1800 °C for 5 s. All samples were run in triplicate. For TEM analysis, the cells were collected after 24 h of E-GNP (50 µg/mL) or C-GNPs (50 µg/mL) treatment and washed two times with PBS. The cells were pelleted by centrifugation and fixed overnight in 3% glutaraldehyde. After osmium tetroxide fixation, ethanol dehydration, and infiltration with propylene oxide/EPON 812, the pellet was embedded in EPON 812. After embedding, thin sections were cut, mounted on copper TEM grids, and examined on Philips CM100 TEM (Eindhoven, Netherlands).

*Laminin receptor blocking study:* RWPE1, MCF-10A, PC3, and MDA-MB-231 cells (2.5 × 10^5^ cells/well) were seeded in six-well plates and incubated with anti-laminin receptor antibody, MLuC5 (5 µg/mL) for 1 h. Thereafter, cells were treated with E-GNPs (50 µg/mL) or C-GNPs (50 µg/mL) for 3 h and lysed by sonication. Released nanoparticles were centrifuged, washed, diluted in 1% HNO_3_ and subjected to AAS analysis with a graphite furnace atomic absorption spectrometer to determine their concentration. The cells which were not pre-incubated with MLuC5 were used as controls.

*In vitro drug release study:* E-GNPs (10 mL of 1 mg/mL suspension) or equivalent amount of free EGCG (1.59 mM; 10 mL) was placed in a dialysis bag (3.5 kDa molecular weight cutoff). The dialysis bags were suspended separately in 100 mL of phosphate buffered saline (PBS, pH 7.4) with gentle stirring at 37 ± 0.5 °C. Samples of 1 mL were collected from the incubation medium at predetermined time intervals and the removed incubation medium was replaced with equal volume of fresh PBS to maintain sink condition. The collected samples were analyzed by spectrophotometry to quantify EGCG release behavior.

*Measurement of apoptosis:* PC3 and MDA-MB-231 cells (2.5 × 10^5^ cells/well) were exposed to EGCG (11.9 µM), C-GNP (50 µg/mL), or E-GNP (50 µg/mL) for 72 h. After treatment, cells were stained with 7-amino-actinomycin (7-AAD) and PE Annexin V using the PE Annexin V apoptosis detection kit and analyzed by flow cytometry as per manufacturer’s instructions.

*Nuclear and cytoplasmic fractionation:* The cytoplasmic and nuclear extracts were prepared using the nuclear extract kit (Active Motif, Carlsbad, CA, USA) as described previously [31].

*Immunoblot analyses:* Immunoblotting was performed as previously described [32]. Briefly, protein samples (60 µg) were resolved on 10% polyacrylamide gels and transferred to PVDF membrane. The membrane was blocked with 5% milk for 1 h and exposed to the specific primary antibodies and incubated overnight at 4 °C. Next, following washing with TBST buffer (3 times), the membrane was incubated for 1 h with horseradish peroxidase-conjugated secondary antibody. Bands were visualized using ECL plus western blotting substrate kit (Thermo Scientific) with an LAS-3000 image analyzer (Fuji Photo Film Co., Tokyo, Japan).

*NF-κB transcriptional activity assay:* PC3 and MDA-MB-231 (2.5 × 10^5^) cells were grown overnight in a six-well plate and transiently transfected with 1 μg of NF-κB-luciferase reporter construct (i.e., pGL4.32 [*luc*2P/NF-κB -RE/Hygro]) and 0.5 μg of Renilla luciferase control reporter vector (pRL-TK). After 24 h of transfection, cells were treated with EGCG (11.9 µM), C-GNP (50 µg/mL), or E-GNP (50 µg/mL) for 24 h and the cell lysates were collected in passive lysis buffer. Luciferase activity was measured using the Dual Luciferase Assay System (Promega, Madison, WI, USA) according to the manufacturer’s protocol.

*Statistical analysis:* Wherever suitable, the experiments were performed three times, and data expressed as mean ± SD. Wherever appropriate, the data were also subjected to unpaired two-tailed Student’s *t*-test. A value of *p* < 0.05 was considered statistically significant.

## 3. Results

### 3.1. Synthesis and Characterization of C-GNPs and E-GNPs

**Figure 1:**
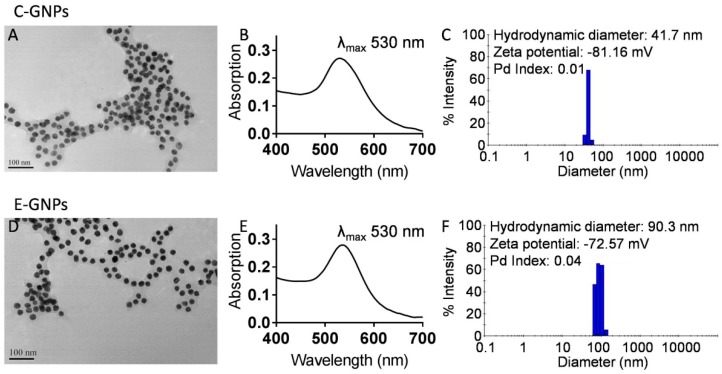
Characterization of citrate-gold nanoparticles (C-GNPs) and EGCG-gold nanoparticles (E-GNPs). (**A**) Transmission electron microscopy (TEM) image of C-GNPs shows that the nanoparticles are spherical in shape and ~25 nm in size. (**B**) The absorption spectrum of C-GNPs showing λ_max_ at 530 nm. (**C**) Dynamic light scattering (DLS) measurement of C-GNPs showing their size distribution, hydrodynamic diameter, and zeta potential. (**D**) TEM image of E-GNPs shows that the nanoparticles are spherical in shape and ~25 nm in size. (**E**) The absorption spectrum of E-GNPs showing λ_max_ at 530 nm. (**F**) Dynamic light scattering measurement of E-GNPs showing their size distribution, hydrodynamic diameter, and zeta potential.

C-GNPs were synthesized by reducing HAuCl_4_·3H_2_O with trisodium citrate (TSC) as reported earlier [21], with some modifications. On the other hand, E-GNPs were synthesized by reducing HAuCl_4_·3H_2_O with EGCG, at room temperature. After synthesis, C-GNPs and E-GNPs were characterized by UV-visible absorption spectroscopy, TEM, and DLS. The UV-visible absorption spectra analysis showed that both the nanoparticles (C-GNPs and E-GNPs) had λ_max_ at 530 nm (Figure 1B,E), indicating that the nanoparticles were of similar size [33]. Further, TEM analysis showed that both the nanoparticles were spherical in shape and ~25 nm in diameter (Figure 1A,D). DLS analysis demonstrated that C-GNPs and E-GNPs had a very low polydispersity index (PD index), which implies uniform size distribution (Figure 1C,F). Further, hydrodynamic diameters of C-GNPs and E-GNPs were 41.7 and 90.3 nm, and their zeta potentials were −81.16 and –72.57 mV, respectively. The amount of citrate or EGCG attached to the surface of C-GNPs and E-GNPS, respectively, was assessed indirectly by quantifying the unloaded citrate or EGCG after the synthesis and subtracting it from the total amount used for the reactions. We observed that 1.2 µg of citrate and 2.35 µg of EGCG were loaded per 1 mg of C-GNPs and E-GNPs, respectively (supplementary materials, Figure S1). In addition, we found that E-GNPs were stable even after 6 months of storage at 4 °C (supplementary materials, Figure S2).

### 3.2. E-GNPs Exhibit Selective and Superior Anticancer Activity

**Figure 2:**
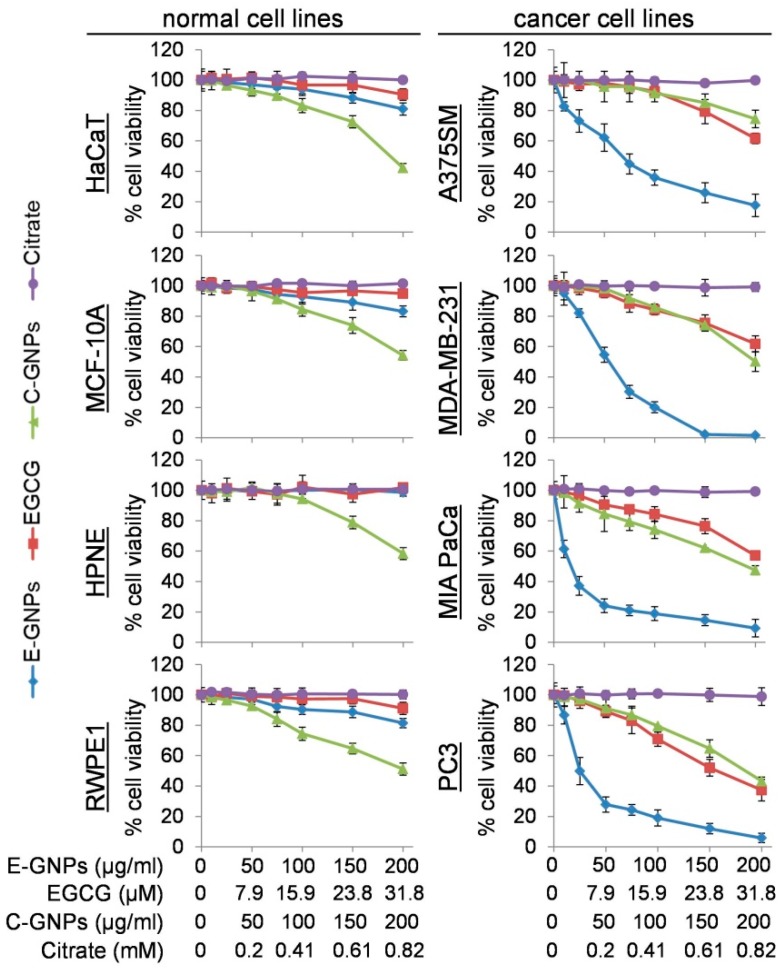
E-GNPs exhibit selective and superior anticancer activity. 5 × 10^3^ noncancerous cells (HaCaT, MCF-10A, HPNE, and RWPE1) and cancerous cells (A375SM, MDA-MB-231, MIA PaCa, and PC3) were seeded in 96-well plates and treated with different concentrations of E-GNPs, epigallocatechin gallate (EGCG), C-GNPs, or citrate for 96 h, and cell viability was determined by WST-1 assay. Data represent the mean of triplicates ± SD.

Antitumor activity of E-GNPs, free EGCG, C-GNPs, and citrate was assessed on four human cancer cell lines (A375SM, MDA-MB-231, MIA PaCa, and PC3). As a control, we also used four non-cancerous (immortalized normal) cell lines (HaCaT, MCF10A, HPNE, and RWPE-1). Cells were treated with increasing concentrations of E-GNPs (0–200 µg/mL), EGCG (0–31.8 µM), citrate (0–0.82 mM), and C-GNPs (0–200 µg/mL) for 96 h and their viability measured by WST-1 assay. Our data demonstrated that E-GNPs caused significantly greater dose-dependent growth inhibition of cancer cells as compared to citrate, EGCG, or C-GNPs (Figure 2). IC_50_ values of E-GNP were found to be 67.6, 54.7, 17.0, and 24.9 µg/mL for A375SM, MDA-MB-231, MIA PaCa and PC3, respectively. At comparable doses of C-GNPs and free EGCG, we observed growth inhibition of 4.3% and 3.8% (A375SM), 3.0% and 5.8% (MDA-MB-231), 4.9% and 2.2% (MIA PaCa), and 2.9% and 4.0% (PC3), respectively. Negligible or low toxicity towards normal cells was reported for free EGCG, citrate, and E-GNPs. In fact, at the highest dose (200 µg/mL), E-GNPs exhibited 19.0%, 16.9%, 1.4%, and 18.5% growth inhibition for HaCaT, MCF10A, HPNE, and RWPE-1, respectively, as compared to 57.7%, 45.8%, 41.6%, and 48.8% for C-GNPs (Figure 2). Citrate exhibited negligible toxicity towards normal and cancer cells. The synergy calculations indicated synergism [combination index (CI) < 1] between EGCG and GNPs in E-GNP nanoformulation with CIs of 0.19 (A375SM), 0.48 (MDA-MB-231), 0.12 (MIA PaCa), and 0.31 (PC3). 

### 3.3. Cancer Cells Internalize E-GNPs More Efficiently Than Normal Cells

**Figure 3:**
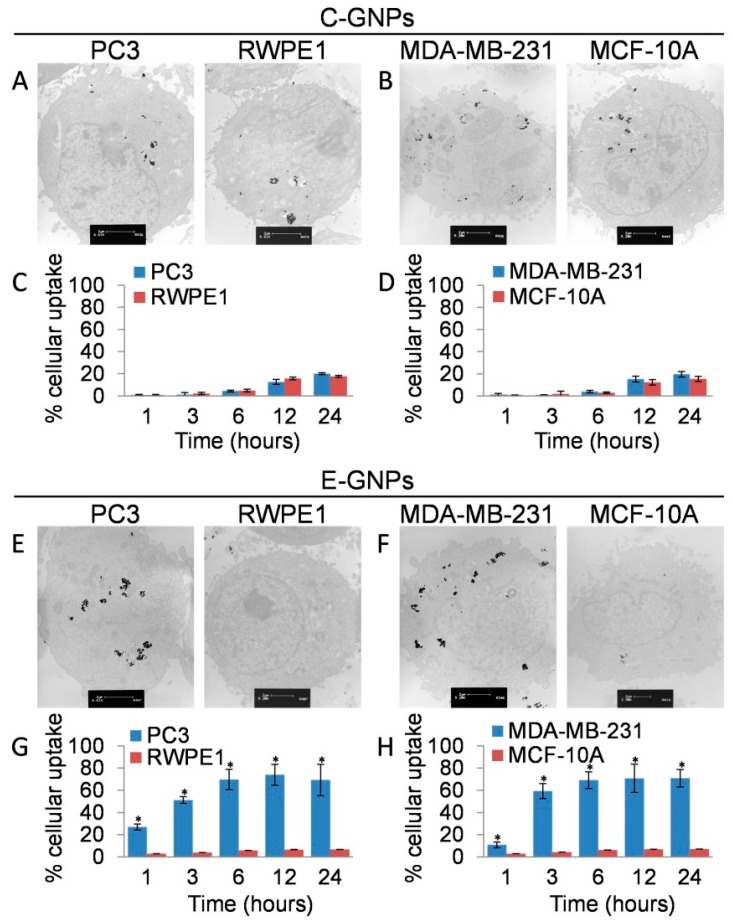
Cancer cells internalize E-GNPs more efficiently. TEM images of PC3 and RWPE1 cells after 24 h treatment with C-GNPs (**A**) and E-GNPs (**E**) at 50 µg/mL concentration. TEM images of MDA-MB-231 and MCF-10A cells after 24 h treatment with C-GNPs (**B**) and E-GNPs (**F**) at 50 µg/mL concentration. Atomic absorption spectroscopy (AAS) analysis data from PC3 and RWPE1 cells show time-dependent cellular uptake of C-GNPs (**C**) and E-GNPs (**G**). AAS analysis data from MDA-MB-231 and MCF-10A cells shows time-dependent cellular uptake of C-GNPs (**D**) and E-GNPs (**H**). Bars represent the mean of triplicates ± SD, * *p* ˂ 0.05.

We analyzed the cellular uptake of C-GNPs and E-GNPs in normal (RWPE1 and MCF-10A) and cancerous (PC3 and MDA-MB-231) cell lines using TEM and AAS analysis after treating the cells with 50 µg/mL of C-GNPs or E-GNPs for 24 h. TEM analysis revealed that there was no substantial difference in the uptake of C-GNPs between normal and cancerous cells, whereas E-GNPs were taken up by cancer cells more efficiently compared to normal cells (Figure 3A,B,E,F). Further, no nanoparticle clustering was observed in either of the nanoparticles even after cellular uptake (supplementary materials, Figure S3). Moreover, time-dependent uptake studies by AAS analysis indicated that C-GNPs were taken up slowly and less efficiently by all the cell lines (Figure 3C,D). Importantly, even after 24 h incubation, internalization of C-GNPs by different cell lines ranged only between 15.4–20.1%. On the other hand, more than 50% of E-GNPs were taken up by both the cancer cell types within the first 3 h of incubation (Figure 3G,H). Furthermore, after 24 h of treatment, 69.3% (PC3) and 70.9% (MDA-MB-231) of E-GNPs were internalized (Figure 3G,H). Interestingly, internalization of E-GNPs was much less in both the normal cell lines [RWPE1 (6.6%) and MCF-10A (7.0%)] even after 24 h of incubation (Figure 3G,H). These results indicate that there is a ~10-fold difference in the uptake of E-GNPs by normal and cancerous cells.

### 3.4. Laminin Receptor Blocking Reduces Uptake of E-GNPs by Cancer Cells

**Figure 4:**
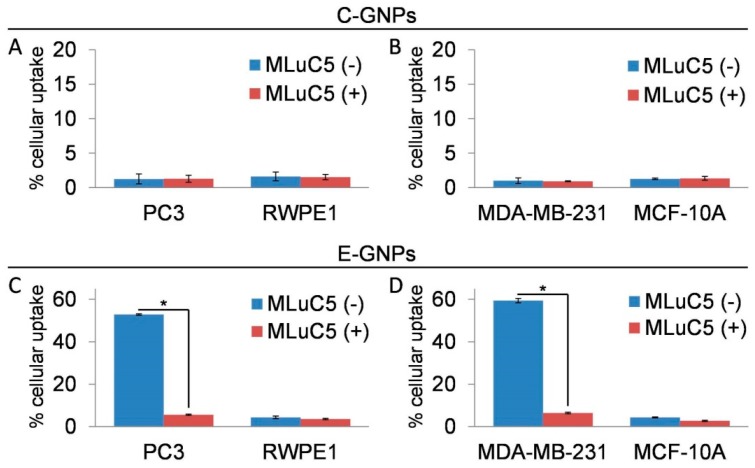
Laminin receptor blocking significantly reduces uptake of E-GNPs by cancer cells. Atomic absorption spectroscopy (AAS) analysis data from PC3 and RWPE1 cells showing the effect of laminin receptor blocking by MLuC5 antibody on cellular uptake of C-GNPs (**A**) and E-GNPs (**C**). AAS analysis data from MDA-MB-231 and MCF-10A cells showing the effect of laminin receptor blocking by MLuC5 antibody on cellular uptake of C-GNPs (**B**) and E-GNPs (**D**). Bars represent the mean of triplicates ± SD, * *p* ˂ 0.05.

EGCG is reported to serve as a ligand for the laminin receptor [12,13,14,15]. To determine if this was the cause of the efficient and selective uptake of E-GNPs by cancer cells, we first analyzed the expression of laminin receptors in cancerous and non-cancerous cell lines used in our study. We observed that all the cancer cell lines had significantly greater laminin receptor expression, as compared to non-cancerous cells (supplementary materials, Figure S4). Next, we performed a laminin receptor blocking experiment to determine their role in promoting uptake of E-GNPs by cancer cells. The data show that laminin receptor blocking by MLuC5 antibody led to a drastic decrease in cellular uptake of E-GNPs by cancer cells (PC3 and MDA-MB-231), whereas no significant difference was observed in the case of normal cells (RWPE1 and MCF-10A) (Figure 4A). Similarly, we did not observe any effect of laminin receptor blocking on cellular uptake of C-GNPs in all the cell lines tested (Figure 4B). 

### 3.5. E-GNP Nanoformulation Releases EGCG in a Sustained Fashion

**Figure 5:**
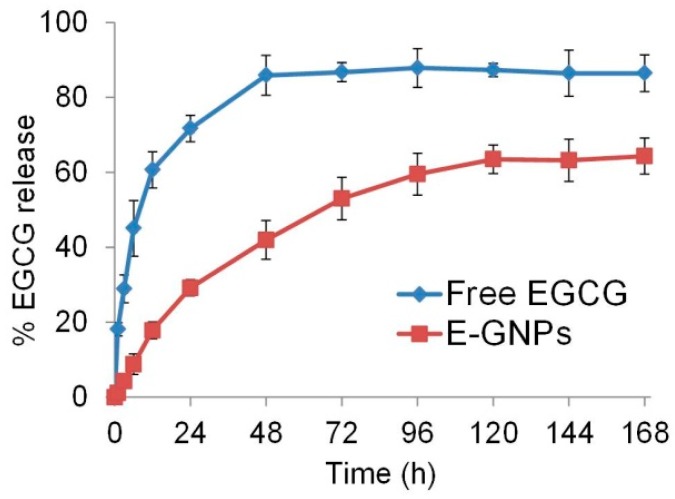
E-GNP nanoformulation releases EGCG in a sustained fashion. The time-dependent drug release behaviors of E-GNPs and free EGCG were quantified by spectrophotometry. The graph clearly shows sustained release of EGCG from E-GNPs nanoformulation. Data represent the mean of triplicates ± SD.

Slow and sustained release of a drug can lead to enhanced potency [34,35]. Therefore, we examined if the EGCG in E-GNPs nanoformulation was released slowly as compared to its free form in solution. EGCG release was monitored by dialysis, followed by quantification of released drug by spectrophotometry. We observed that 41.9% of the EGCG was released into the incubation medium from E-GNPs in 48 h, and the sustained release continued for 5 days (Figure 5). In contrast, we observed that 85.9% of free EGCG was released from the dialysis bag into the incubation medium within 48 h, and reached a plateau thereafter. These data suggest that E-GNP nanoformulation releases EGCG in a sustained fashion.

### 3.6. E-GNPs Induce Apoptosis in Cancer Cells more Potently than C-GNPs

**Figure 6:**
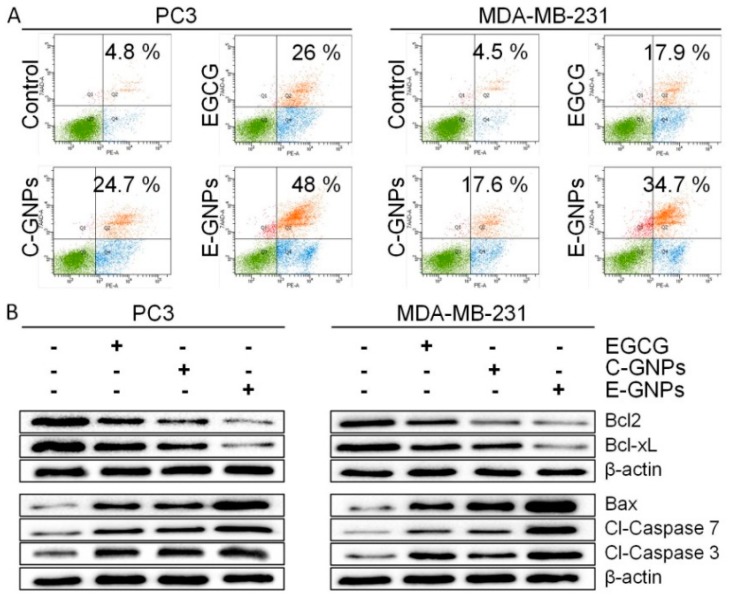
E-GNPs induce apoptosis in cancer cells. (**A**) The number of apoptotic cells after treatment with EGCG, C-GNPs, and E-GNPs was determined by Annexin V binding assay, using flow cytometry. (**B**) Total protein was isolated and immunoblot analysis was performed to analyze the expression of BCL2, BCL-xL, Bax, cleaved-caspase 7, and cleaved caspase 3. β-actin was used as loading control.

To further explore the potential mechanisms by which E-GNPs induce growth inhibitory effects in cancer cells, we measured the extent of apoptosis after treatment with C-GNPs, E-GNPs, and free EGCG for 24 h. Treated PC3 and MDA-MB-231 cells were stained with Annexin V and subjected to flow cytometry. We observed that EGCG induced 26% (PC3) and 17.9% (MDA-MB-231) apoptosis, whereas C-GNPs induced 24.7% (PC3) and 17.6% (MDA-MB-231) apoptosis (Figure 6A). Importantly, more apoptosis was observed in PC3 (48.0%) and MDA-MB-231 (34.7%) cells when they were treated with E-GNPs (Figure 6A). In addition, we also studied the expression of apoptosis-related proteins (BCL2, BCL-xL, Bax, cleaved caspase 7, and cleaved caspase 3), by immunoblotting, in PC3 and MDA-MB-231 cells treated with EGCG, E-GNPs, and C-GNPs for 24 h. We observed a drastic reduction in the expression of anti-apoptotic proteins BCL2 and BCL-xL in E-GNPs treated cells as compared to those treated with EGCG and C-GNPs (Figure 6B). Similarly, treatment with E-GNPs resulted in the enhanced expression of Bax, cleaved caspase 7 and cleaved caspase 3 in both PC3 and MDA-MB-231 cells in comparison to EGCG and C-GNPs (Figure 6B).

### 3.7. E-GNPs Abrogate Nuclear Translocation and Transcriptional Activity of NF-κB in Cancer Cells More Effectively Than Free EGCG or C-GNPs

**Figure 7:**
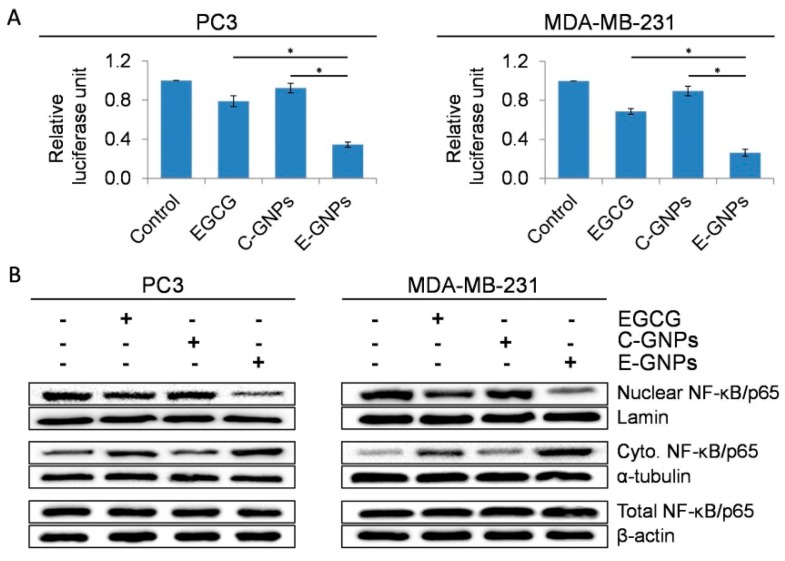
E-GNPs abrogate nuclear translocation and transcriptional activity of NF-κB in cancer cells. (**A**) The NF-κB-luciferase reporter construct and Renilla luciferase control reporter vector transfected PC3 and MDA-MB-231 were treated with EGCG (11.9 µM), C-GNP (50 µg/mL), or E-GNP (50 µg/mL) and NF-κB transcriptional activity was examined after 24 h using the Dual Luciferase Assay System. (**B**) After treatment with EGCG, C-GNPs, and E-GNPs, total protein was isolated and immunoblot analysis was performed to analyze the expression of nuclear NF-κB, cytosolic NF-κB, and total NF-κB. Lamin, α-tubulin, and β-actin were used as loading controls respectively. Bars represent the mean of triplicates ± SD, * *p* ˂ 0.05.

NF-κB is known to regulate the expression of survival-associated genes, including those of members of the BCL2 family [31]. Moreover, EGCG has also been shown to reduce the activity of NF-κB [36]. Therefore, we sought to study and compare the effect of EGCG, C-GNP, and E-GNP on the activity of NF-κB. Transcriptional activity of NF-κB-responsive promoter–reporter was analyzed by dual-luciferase reporter assay in transfected PC3 and MDA-MB-231 cells treated with EGCG, C-GNP, or E-GNP (Figure 7A). In both the cell lines, E-GNPs caused excellent suppression of NF-κB transcriptional activity (65.5% in PC3 and 73.7% in MDA-MB-231) as compared to free EGCG (21.1% in PC3 and 31.3% in MDA-MB-231) and C-GNPs (7.7% in PC3 and 10.3% in MDA-MB-231). The NF-κB dimers are present in the cytoplasm in an inactive form and upon activation, they translocate into the nucleus, where they bind to specific DNA motifs in the promoter/enhancer regions of target genes and activate their transcription [37]. To examine if these changes in NF-κB transcriptional activity resulted from altered sub-cellular localization, we isolated nuclear, cytoplasmic and total protein fractions from PC3 and MDA-MB-231 cells treated with EGCG, C-GNPs, or E-GNPs for 24 h. Accumulation of NF-κB/p65 in the nucleus and cytoplasm or any changes in the overall expression was determined by immunoblot assay. Our data show that E-GNPs cause a significantly greater reduction in the nuclear accumulation of NF-κB/p65 in cancer cells in comparison to EGCG and C-GNP (Figure 7B). No change in the level of total NF-κB/p65 was detected after the treatment with EGCG, E-GNPs or C-GNPs (Figure 7B).

## 4. Discussion

This study presents data to support the superior antitumor efficacy of EGCG-synthesized GNPs, as compared to conventional GNPs, due to potential synergistic interactions. EGCG, a bioactive polyphenol found in green tea [6], is shown to cause growth inhibition and apoptosis in several cancers [8,9]. However, its therapeutic applications are limited due to its poor stability and systemic bioavailability [16,17,18]. On the other hand, nanoparticles serve as drug carriers and provide protection to the loaded drugs [8,38]. More importantly, some nanoparticles, like GNPs, also possess intrinsic anticancer activity [23,24,25,26,27] and thus, combining the two may yield greater therapeutic benefit due to potential synergistic interactions.

We synthesized C-GNPs and E-GNPs by reducing Au(III) chloride with citrate and EGCG, respectively. Although our TEM data showed that both C-GNPs and E-GNPs had comparable shape and size distribution, the average hydrodynamic diameter of E-GNPs was higher than that of C-GNPs, possibly due to the difference in their surface coating. EGCG, upon reducing Au^3+^ to Au^0^, becomes physically adsorbed onto the GNP surface. Further, the high density of free -OH groups in EGCG could lead to the formation of a thick layer of EGCG supramolecular assemblies on the nanoparticle core, due to extensive inter- and intramolecular hydrogen bonding [39,40]. It is worth noting, however, that some amount of citrate/EGCG would have oxidized during GNP synthesis and indirect measurement of adsorbed citrate/EGCG may not provide us the precise estimate. Hence, in our comparison studies, we used the free citrate/EGCG amount that was comparable to the highest possible amount of unoxidized citrate/EGCG that could have been possibly adsorbed on the GNPs. Despite this, we observed that E-GNPs possessed superior anticancer activity in all the cancerous cell lines tested in comparison to free EGCG or C-GNPs. This increased anticancer activity of E-GNPs could be due to the increased stability of EGCG in the nanoformulation [41]. Furthermore, EGCG in E-GNP nanoformulation might have escaped efflux, as it is known that EGCG is actively pumped out of cancer cells in its free form [42,43] and nanoformulations of drugs can evade drug efflux [44,45,46]. Moreover, the superior anticancer activity of E-GNPs over C-GNPs could be due to the better cellular uptake of E-GNPs. All cancer cell lines used in our study exhibit an overexpression of laminin receptor. Since EGCG is shown to bind to laminin receptor [12,13,14], this might be an important mechanism for increased and selective cellular uptake of E-GNPs by cancer cells as compared to C-GNPs. To test this, we have done laminin receptor blocking studies and found that EGCG helps in cancer cell-specific uptake of E-GNPs by binding to overexpressed laminin receptors on these cells. As well as selective and increased uptake of E-GNPs by cancer cells, sustained release of EGCG from E-GNP nanoformulation could have resulted in this increased anticancer efficacy of E-GNPs. Additionally, the difference in hydrodynamic diameter of C-GNPs and E-GNPs might also have played a role in the differential uptake of the nanoparticles as reported by others in prior studies [47,48,49]. 

Apoptosis is a cellular suicide mechanism in which cells are programmed to die upon receiving an apoptotic stimulus to eliminate unwanted or unhealthy cells. However, cancer cells have developed mechanisms to resist apoptotic cell death through overexpressed of survival proteins. Considering the fact that both GNPs and EGCG induce apoptosis in cancer cells [26,50,51], we hypothesized that E-GNPs would exhibit superior efficacy. Our study revealed that exposure of PC3 and MDA-MB-231 cells to EGCG, C-GNPs, or E-GNPs induces apoptosis. However, E-GNP nanoformulation was the most efficient. This is likely due to synergistic interactions between EGCG and GNPs (i.e., EGCG stabilization by GNPs and increased uptake of E-GNPs facilitated by EGCG coating) as well as their inherent antitumor activities. We also found that anti-apoptotic proteins were downregulated and pro-apoptotic proteins were upregulated in all the three treatments, with treatment with E-GNPs showing a better outcome. A high Bax/Bcl-2 ratio can cause mitochondrial membrane depolarization, leading to the release of intermembrane space proteins into the cytosol, where they promote caspase-dependent apoptosis [52,53,54]. 

Several transcription factors, including NF-κB, are suggested to regulate the expression of Bcl-2 and Bcl-xL in several cancer types [55]. Importantly, EGCG is reported to reduce the nuclear accumulation of NF-κB by downregulating IKK which activates NF-κB by phosphorylating IκB [36]. NF-κB, a member of the Rel family of proteins, consists of closely related transcription factors that bind to the κB site, a common sequence motif in DNA [36,56]. NF-κB plays an important role in the regulation of multiple cellular processes including cell survival, proliferation, and apoptosis at the gene level [56]. The role of NF-κB in the pathobiology of several cancers has been explored well [57,58]. It has been established that cancer cells counteract the induction of apoptosis by the activation of NF-κB [59,60]. The activated NF-κB is translocated from the cytoplasm to the nucleus, where it induces the expression of numerous genes involved in the suppression of apoptosis, including Bcl-2 and Bcl-xL [55]. In this context, our data showing reduced nuclear accumulation and transcriptional activity of NF-κB is quite significant. Remarkably, the negative regulation of NF-κB by E-GNPs is significantly greater than that by free EGCG or C-GNPs. Further, C-GNPs did not show much effect on the NF-ĸB activity; however, the expression of apoptosis-associated proteins was regulated by C-GNPs. Notably, EGCG does have some repressive effect on NF-ĸB activity. We believe that the reason for synergism between EGCG and GNPs in E-GNPs is due to EGCG stabilization by GNPs and increased uptake of E-GNPs facilitated by EGCG through binding with laminin receptor. As well as increased stability and cellular uptake of E-GNPs, the simultaneous targeting of multiple signaling pathways involved in the activation of NF-κB by E-GNP nanoformulation could have caused this effect, which remains to be investigated in more precise detail in subsequent studies [61].

## 5. Conclusions

In conclusion, we report a synergistic anticancer activity between GNPs and EGCG in E-GNP nanoformulation. The increased cellular uptake or retention of E-GNPs and improved stability of EGCG in the nanoformulation might be the basis for this superior anticancer activity of E-GNPs. Overall, the data suggest that E-GNPs have great potential in the treatment of multiple types of cancer. A clear understanding of upstream and downstream events involved in the superior efficacy of E-GNPs is warranted to gain mechanistic insight and advance its potential for human applications.

## Supplementary Materials

The following are available online at https://www.mdpi.com/2079-4991/9/3/396/s1. Figure S1: Quantification of EGCG loading on GNP; Figure S2: E-GNPs stability studies; Figure S3: Representative TEM images at high magnification (28,500×); Figure S4: Laminin receptor expression in cancerous and noncancerous cell lines.
