# Supplementary material for: Epigallocatechin Gallate-Gold Nanoparticles Exhibit Superior Antitumor Activity Compared to Conventional Gold Nanoparticles: Potential Synergistic Interactions
Source: Nanomaterials (Basel). 2019 Mar 8;9(3):396. doi: 10.3390/nano9030396 (PMC6474148; doi:10.3390/nano9030396)
Supplement: Supplementary file 1 [file nanomaterials-09-00396-s001.pdf]

Epigallocatechin Gallate-Gold Nanoparticles Exhibit Superior Antitumor Activity Compared To Conventional Gold Nanoparticles: Potential Synergistic Interactions

Suhash Reddy Chavva, Sachin Kumar Deshmukh, Rajashekhar Kanchanapally, Nikhil Tyagi, Jason William Coym, Ajay Pratap Singh*, Seema Singh*

Supplementary Material

Supplementary Methods

E-GNPs stability studies

After washing and resuspending E-GNPs in DI water, their UV-Vis absorption spectrum was recorded, and they were stored at 4 °C. Their absorption spectrum was recorded intermittently for six months. As shown in (Figure S2), the peak shape or absorption intensity did not change even after 6 months indicating that the nanoparticles are very stable.

RNA isolation and quantitative reverse transcription polymerase chain reaction (qRT-PCR)

Total RNA from cell lines was extracted using TRIzol reagent (Invitrogen), and reverse-transcribed with high capacity cDNA Reverse Transcription Kit (Applied Biosystems). qRT-PCR was performed using cDNA as a template and SYBR Green Master Mix on an iCycler system (Bio-Rad, Hercules, CA, USA) with laminin receptor specific primer pair sets (Forward: TCACTCAGTGGGTTTGATGTG; Reverse: CACAGGACTAGAACACCTG). GAPDH served as an internal control (Forward: ACAACTTTGGTATCGTGGAAGG; Reverse: GCCATCACGCCACAGTTTC). Following thermal conditions were used: cycle 1: 95 °C for 10 min, cycle 2 (40): 95 °C for 10 s and 58 °C for 45 s.

Supplementary figures

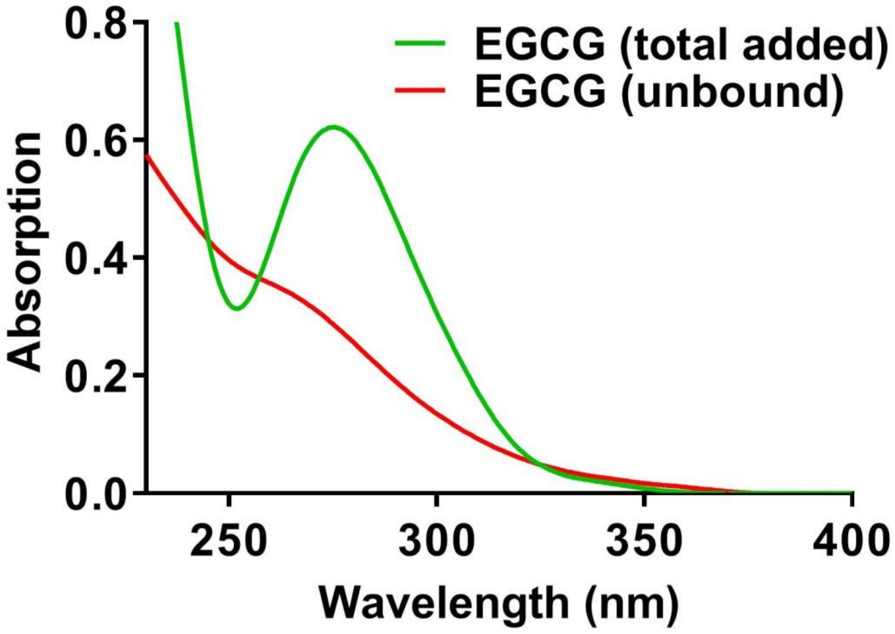

Figure S1: Quantification of EGCG loading on GNP. Absorption spectra of EGCG in the reaction mixture before and after E-GNPs synthesis.

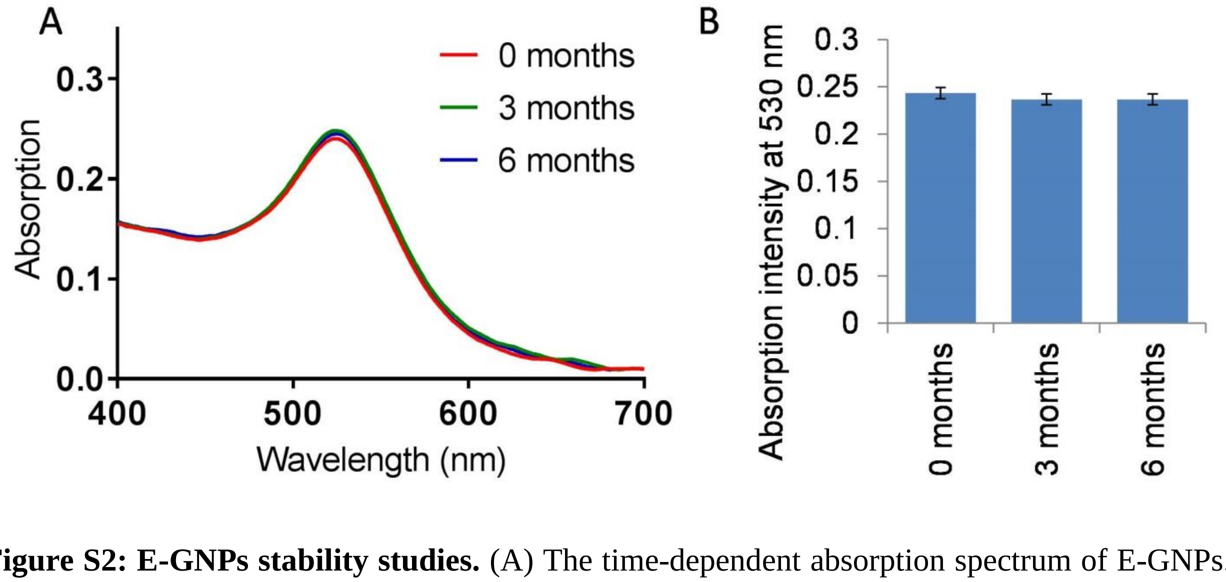

Figure S2: E-GNPs stability studies. (A) The time-dependent absorption spectrum of E-GNPs. (B) E-GNPs absorption intensity at 530 nm. Data represent the mean of triplicates ± SD.

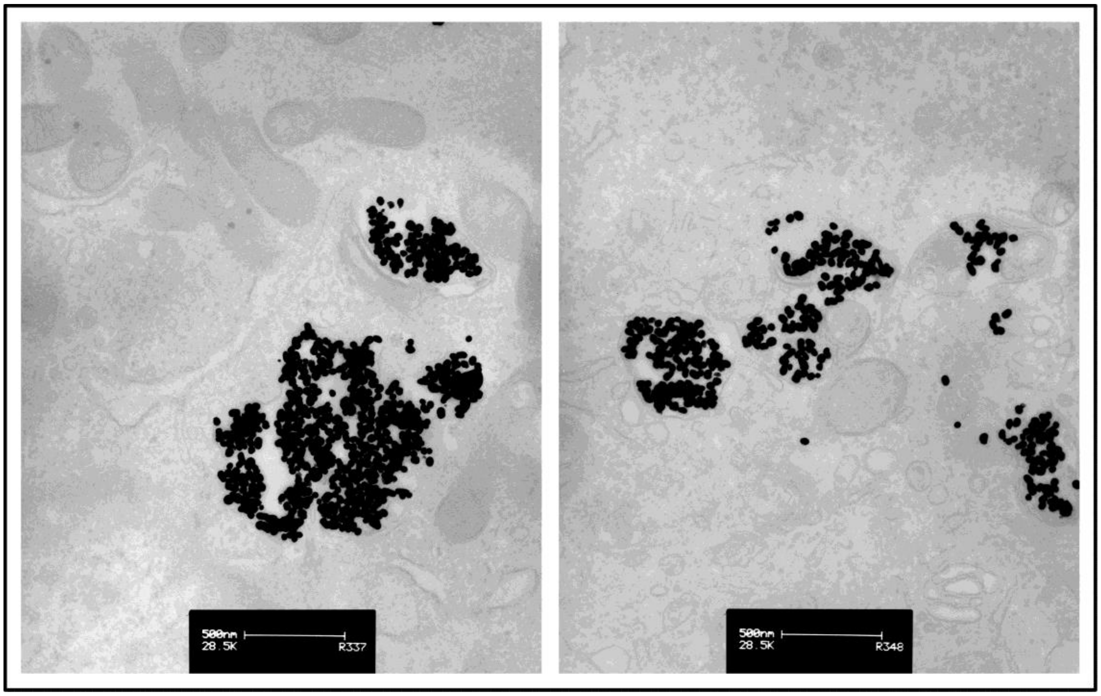

Figure S3: Representative TEM images at high magnification (28500X).

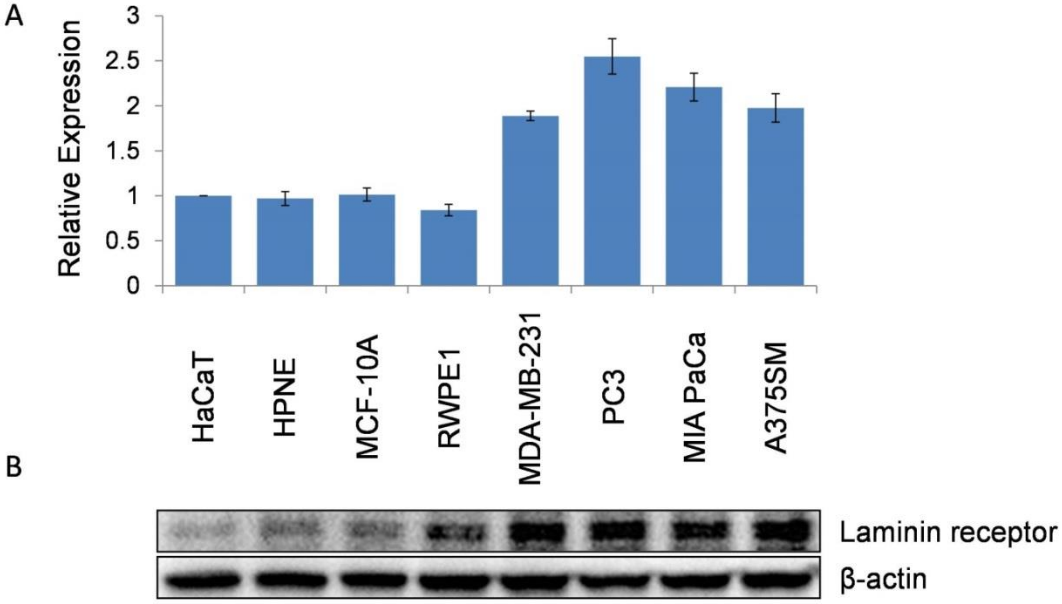

Figure S4: Laminin receptor expression in cancerous and noncancerous cell lines. (A) PCR data. Data represent the mean of triplicates ± SD. (B) Western blot.
